# Decoding the neural dynamics of everyday prospective remembering: a hidden Markov model approach

**DOI:** 10.3389/fnhum.2025.1686657

**Published:** 2026-02-04

**Authors:** Stefano Vicentin, Davide Buzzi Reschini, Paola Santacesaria, Lisa Toffoli, Sara Zago, Giorgio Arcara, Giorgia Cona

**Affiliations:** 1 Department of General Psychology, University of Padua, Padua, Italy; 2 Department of Neuroscience, Biomedicine and Movement Sciences, University of Verona, Verona, Italy; 3 Padova Neuroscience Center, Padua, Italy; 4 IRCCS San Camillo Hospital, Venice, Italy

**Keywords:** prospective memory, electroencephalography, hidden Markov model, pseudo-naturalistic paradigm, dorsal attention network, default-mode network

## Abstract

**Introduction:**

Prospective memory (PM)—the ability to form, maintain, and execute delayed intentions—is essential for everyday functioning. Traditionally, PM paradigms relied on repetitive tasks and focused on transient post-stimulus activity, overlooking the sustained neural processes supporting intention maintenance.

**Methods:**

High-density EEG data were recorded during a naturalistic PM paradigm simulating everyday activities (preparing a meal while watching TV), comprising three conditions: naturalistic viewing, event-based PM, and time-based PM. Using individual MRI and hidden Markov modeling (HMM), brain activity was studied at the source level and splitted into an optimal number of states.

**Results:**

The HMM analysis identified 6 brain states. Among them, State 3 was characterized by activations over regions of the dorsal attention network (DAN) and was more prominent during the timebased PM task, consistently with the DAN role in sustained attention and time monitoring. State 6, involving core regions of the default mode network (DMN), showed longer inter-activation intervals, suggesting a role in transient and sporadic processes (intention retrieval). Crucially, efficient time checks positively correlated with time spent in these two brain states, linking them to PM accuracy.

**Discussion:**

These findings suggest complementary roles of DAN and DMN regions in prospective remembering—continuous monitoring versus retrieval—and demonstrate how combining HMM with naturalistic paradigms offers new insights into the neural dynamics underlying real-world intention maintenance.

## Introduction

In daily life, we frequently plan actions that cannot be executed immediately but must be carried out at specific future moments or at the occurrence of specific events. This ability, known as prospective memory (PM), encompasses the processes of encoding an intention (encoding phase), retaining it in memory over a delay period (maintenance phase), and retrieving it when the appropriate condition occurs (retrieval and execution phases; Brandimonte et al., 1996; McDaniel and Einstein, 2007). During encoding, the intention is associated with a specific event or moment, labeled as the *PM cue*, whose appearance will trigger the retrieval of the intention from memory (Kliegel et al., 2011; Kvavilashvili and Ellis, 1996). Intentions can be associated with specific events, such as remembering to stop by the gas station on the way home (event-based PM task) or specific moments in the future, like remembering to attend a meeting at noon (time-based PM task; Kliegel et al., 2000).

Early PM research was conducted in real-life contexts, employing tasks such as remembering to call researchers at prearranged times or completing daily diaries (Crovitz and Daniel, 1984; Meacham and Leiman, 1975). While these studies provided valuable insights into everyday PM functioning, they suffered from low reproducibility, uncontrolled external factors (e.g., reminders, distractions, non-compliance), and the impossibility of examining the neural mechanisms of prospective remembering. To overcome these limitations, Einstein and McDaniel (1990) introduced the first controlled laboratory paradigm for PM. Subsequent approaches standardized several aspects of task design—such as the low frequency of PM cues, the use of a baseline condition, and the assessment of intention maintenance costs by comparing PM and baseline trials (Anderson et al., 2019; Hicks et al., 2005). This shift from ecological validity to experimental control allowed systematic investigation of the cognitive and neural mechanisms underlying PM.

Within this framework, neuroimaging and electrophysiological studies identified several regions and networks contributing to PM, revealing that it depends on the dynamic coordination of distributed neural systems. The anterior prefrontal cortex, posterior parietal cortex, and hippocampus play key roles in intention maintenance and PM cue detection (Burgess et al., 2001, 2011; Martin et al., 2007). Large-scale functional networks—including the dorsal attention network (DAN), ventral attention network (VAN), and default mode network (DMN)—have been shown to contribute differently depending on the PM phase and task demands. The DAN is typically associated with top-down attentional control and external monitoring, while the VAN supports stimulus-driven attentional capture (Corbetta and Shulman, 2002; Vossel et al., 2014). Accordingly, PM studies have found that the DAN is primarily engaged during intention maintenance, reflecting sustained monitoring, whereas the VAN is more active during retrieval, reflecting cue-related reorientation (Cona et al., 2015, 2023). The DMN, a network implicated in internally oriented cognition (Buckner et al., 2008), shows enhanced activity during tasks with high maintenance load, whereas the DAN exhibits greater activation under increased monitoring demands (Beck et al., 2014). These findings were found both in the maintenance and the retrieval phases (Cona et al., 2020; Vicentin et al., 2025), stressing the importance of the DAN in top-down mechanisms oriented toward detecting the PM cue and the DMN in internally oriented cognition to maintain and recall the intention(s). Indeed, successful prospective remembering relies on the continuous interplay between attentional and mnemonic processes, engaging similar mechanisms to those that support episodic encoding and retrieval (Madore and Wagner, 2022; Schwartz et al., 2025). Recent EEG work also indicates that fluctuations in attentional intensity and selectivity modulate mnemonic representations within frontoparietal and default-mode regions (Madore et al., 2020; Tran et al., 2025). Finally, event-based and time-based PM tasks have been associated with distinct neural patterns: the former engaging occipito-parietal regions involved in visual processing, and the latter recruiting dorsolateral prefrontal and parietal areas linked to self-initiated monitoring and time estimation (Gonneaud et al., 2014).

Although controlled laboratory paradigms have been instrumental in uncovering PM mechanisms, their limited ecological validity remains a major concern. A well-known example is the age–PM paradox, which describes the tendency of older adults to perform worse than younger adults in laboratory settings but to outperform them in naturalistic contexts (Rendell and Thomson, 1999; Schnitzspahn et al., 2020). To bridge this gap, a growing body of research has focused on enhancing the naturalness of lab-based PM paradigms, building on the assumption that naturalistic investigation exists along a continuum rather than as a dichotomy (Kvavilashvili and Ellis, 2004). These efforts involve the use of more realistic techniques, such as virtual reality (Mioni et al., 2023; Trawley et al., 2017), as well as more naturalistic stimuli and task designs simulating real-world contexts and activities—for example, preparing the table for breakfast (Altgassen et al., 2015; Schmitter-Edgecombe et al., 2012) or watching movies while monitoring the passage of time (Mioni et al., 2012; Mioni and Stablum, 2014). Among other paradigms, movie viewing has been increasingly employed in cognitive neuroscience as a means to study brain activity in realistic yet controlled conditions (Saarimäki, 2021; Virk et al., 2024). The use of movie excerpts—either presented alone or as a background/ ongoing activity—has been termed *naturalistic viewing* (Meer et al., 2020; Sonkusare et al., 2019).

As previously mentioned, movie-based paradigms have also been introduced in behavioral PM research, typically as ongoing tasks in time-based PM designs. Building on these approaches, combining movie watching with naturalistic PM instructions (requiring intention maintenance over longer, realistic intervals) and advanced analysis methods could enhance the ecological validity of findings on the neural mechanisms supporting PM.

To investigate dynamic neural mechanisms over extended, continuous periods, hidden Markov modeling (HMM) provides a powerful analytical framework (Vidaurre et al., 2018). HMM is an unsupervised machine learning technique that can identify recurring, quasi-stable patterns of coordinated brain activity, each characterized by distinct network states and their temporal dynamics (Obermaier et al., 2001; Williams et al., 2018). HMM has been successfully applied to different types of continuous EEG recordings (Toffoli et al., 2024; Zdorovtsova et al., 2023), demonstrating its versatility in capturing brain dynamics across different cognitive contexts. Each pattern—or state— is defined by a set of metrics representing the percentage of time spent in that state (*fractional occupancy, FO*), the average duration of these visits (*state lifetime, LT*), the average interval between consecutive visits (*interval time, IT*), and the frequency of transitions across states (*switching rate, SR*). Overall, HMM provides a powerful framework for characterizing how brain states emerge and transition over time, offering insights into both sustained neural activations (intention maintenance) and rapid neural shifts (intention retrieval).

### The present study

The aim of this project is to investigate the neural correlates of PM in more naturalistic conditions by identifying PM-related spatiotemporal patterns of brain activity within regions broadly consistent with large-scale functional networks—such as the DAN, the VAN, and the DMN—using HMM. To achieve this, we developed a pseudo-naturalistic paradigm comprising three conditions: a naturalistic viewing condition (movie excerpt presented alone), an event-based PM task (movie excerpt + detection of a PM cue), and a time-based PM task (movie excerpt + monitoring the passage of time). Prospective instructions were designed to simulate everyday activities. High-density EEG data were continuously recorded across the three conditions, along with behavioral measures (accuracy, response times, and time-check frequency) to assess performance and task engagement. Sensor-level EEG data were analyzed in a previous study (Santacesaria et al., 2025), which revealed distinct spatial and spectral patterns differentiating the two PM conditions from the Naturalistic Viewing baseline—specifically, enhanced fronto-temporal high-beta activity during time-based PM and occipito-parietal theta–alpha modulations during event-based PM. Building on these findings, the present study applies hidden Markov modeling (HMM) to examine the temporal organization and source-level dynamics associated with the two types of prospective remembering, decomposing EEG data into transient neural states reflecting coordinated large-scale activity. We hypothesize that these states will reveal condition-specific differences in the engagement of regions associated with PM and external/temporal monitoring. Specifically, we predict the emergence of a state characterized by activation of DAN regions—critical for sustained top-down attentional control—with significantly higher FO (i.e., proportion of time spent in the state) in the PM conditions compared to the naturalistic viewing condition, especially during the time-based PM task due to the demand for constant time monitoring. Similarly, states involving regions linked to time estimation processes are expected to be more involved during the time-based condition, while states displaying activity associated with DMN and VAN regions will present longer IT (i.e., intervals between state reactivations), reflecting their occasional engagement during intention retrieval. Finally, we explored associations between HMM-derived metrics and behavioral measures, predicting that greater time spent in DAN-like states would correlate with more efficient PM performance, particularly in time monitoring. By leveraging HMM to uncover hidden neural dynamics, this study aims to advance our understanding of how large-scale brain networks support PM in more naturalistic paradigms, shedding light on the temporal architecture of intention maintenance and retrieval.

## Materials and methods

### Participants

Thirty-one healthy young adults were recruited for the study, all of whom provided written informed consent. The study received approval from the Ethics Committee (protocol no. 2021.24) of the IRCCS San Camillo Hospital (Venice, Italy) and was conducted in line with the Helsinki Declaration principles. EEG data from four participants were excluded after visual inspection due to excessive muscular and electrical artifacts. The final sample thus comprised 27 participants (age = 30.56 ± 5.02 years; years of education = 20.48 ± 3.48; 16 females).

### Experimental paradigm

The experiment was conducted in a room at the Neurophysiology Lab of the IRCCS San Camillo Hospital and lasted approximately 1 h. First, head size was measured to select the appropriate EEG cap, which was then positioned on the participant’s head. Electrode positions were digitized using an iPad-based system to later perform co-registration with individual MRI using the SPOT3D toolbox (Taberna et al., 2019). Hence, participants were seated in a comfortable armchair approximately 60 cm from a 15-inch display (visual angle: 15.7° horizontal, 8.9° vertical). They then received instructions on the structure of the experiment and provided written informed consent.

**Figure 1:**
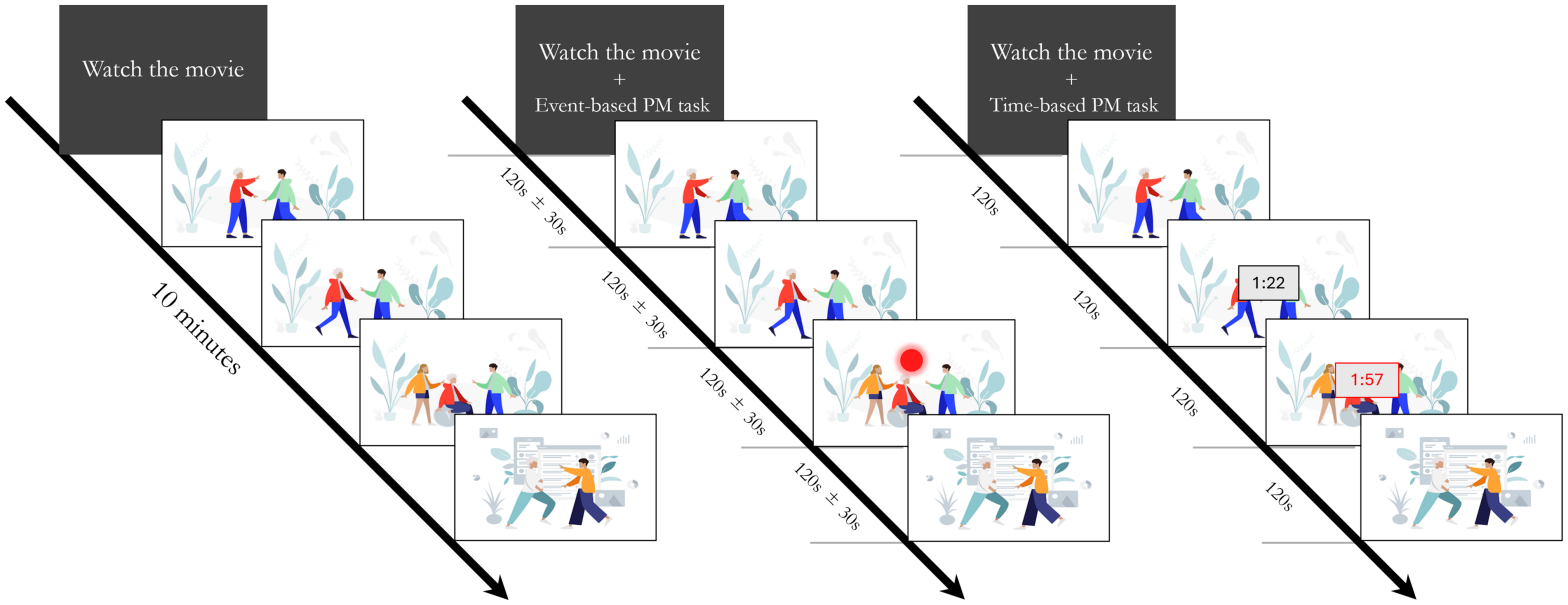
Schematic representation of the three experimental conditions. In the Naturalstic Viewing condition (left), participants passively watched a movie excerpt without additional demands. In the Event-based PM condition (center), participants monitored the movie for the appearance of the red dot (PM cue), which was presented five times at variable intervals (90, 120, or 150 seconds). In the Time-based PM condition (right), participants tracked the passage of time, pressing one key to check the elapsed time (displayed on a black timer) and a second key when they estimated that two minutes had passed. Upon their response, a red clock appeared. Images were created by the authors using open-source illustrations from Humaaans (https://www.humaaans.com; CC BY 4.0).

The experimental paradigm consisted of three conditions: a naturalistic viewing condition and two PM tasks (Figure 1). Each block lasted approximately 10 min, during which one of three movie excerpts from *The Simpsons Movie* (Silverman, 2007) was displayed, in counterbalanced order across participants. Before the experiment began, participants were instructed to relax and imagine they were at home watching TV. The movie excerpts were selected to simulate a typical at-home TV viewing experience. In the naturalistic viewing condition, no additional instructions were provided. This block served as a controlled baseline condition, presenting the same ongoing activity (watching movie excerpts) as in the subsequent PM tasks. By maintaining the same task across conditions, this design enables more direct comparison of neural activity, particularly due to the reduced likelihood of mind-wandering associated with the request to watch a movie compared to traditional resting-state paradigms (Betti et al., 2013; Kvavilashvili and Rummel, 2020). The following two conditions, presented in counterbalanced order across participants, consisted of an event-based and a time-based PM tasks. During these tasks, the movie excerpts served as the ongoing activity, but participants were also instructed to monitor the preparation of a meal in the oven, either by tracking and changing the temperature (event-based PM task) or controlling the timer (time-based PM task). These prospective instructions were inspired by an ecological PM paradigm proposed by Ceci and Bronfenbrenner (1985) and were designed following the guidelines of Einstein and McDaniel (1990) for valid PM paradigms. Specifically, participants were engaged in an ongoing task (movie watching) that they perceived as primary, as they were informed that they would later be asked questions about its content. Second, PM cues occurred infrequently (five times over 10 min) and were embedded within the ongoing trials. Together, these features ensured that participants remained focused on the ongoing activity while maintaining and self-initiating a delayed intention—characteristics that distinguish PM tasks from standard divided-attention paradigms (Einstein and McDaniel, 2019; see also Otani et al., 1997). In the event-based PM condition, participants were instructed to monitor the TV screen for the appearance of a red circle. The occurrence of the circle indicated that the oven’s temperature needed to be adjusted (either increased or decreased, depending on the cue’s position) by pressing the up or down arrow keys. To increase the unpredictability of PM cues, they appeared at irregular intervals (90, 120, and 150 s), counterbalanced across participants. In the time-based PM condition, participants were asked to check the oven every 2 min (120 s) by pressing a key. While watching the movie, participants could monitor the elapsed time up to three times within each 2-min interval by pressing a key to make a digital clock appear in the center of the screen for 2 s. The keys associated with time checking and PM cue response (up and down arrow keys) were counterbalanced across participants. Before the two PM tasks, participants completed a brief practice session to ensure task comprehension. At the end of the experiment, a short questionnaire about the contents of the three movie excerpts was administered to verify that participants paid attention to the ongoing activity. Additionally, participants were asked about their prior familiarity with the movie. The majority (23 out of 27; ~85%) reported having seen it before.

### EEG preprocessing and source estimation

EEG data were acquired using a 256-channel system (Electrical Geodesic Inc.) with a 1,000 Hz sampling rate and considering the average of all electrodes as the reference. Preprocessing was conducted using Brainstorm (Tadel et al., 2011). The EEG signal collected in the three conditions was downsampled to 250 Hz to reduce computational demands and filtered in the [1–30 Hz] frequency band. Bad segments and channels were identified via visual inspection, thus independent component analysis (ICA Infomax; 64 components) was used to remove artifacts clearly related to eye movements, cardiac activity, and motor artifacts. Flat or noisy channels were then interpolated using the spherical spline method.

Individual T1-weighted anatomical MRI images were acquired using a 1.5 Tesla Philips Achieva scanner [repetition time (TR): 8.3 ms, echo time (TE): 4.1 ms, flip angle: 8°, matrix resolution: 288 × 288, slice thickness: 0.87 mm]. MRI preprocess was conducted using Freesurfer (Fischl, 2012) and included skull stripping, subcortical segmentation, cortical surface reconstruction, and generation of smoothed pial and white matter surfaces for the creation of individualized head models. All reconstructed surfaces were visually inspected, and manual edits (control points, pial adjustments) were applied when necessary to ensure anatomical accuracy. To align neurophysiological data with structural images, co-registration was performed using SPM12 by matching digitized EEG electrode positions with individual MRIs via fiducial landmarks (nasion and preauricular points). The forward model was then computed using the boundary element method (BEM; Hall and Hall, 1994), and source estimation was performed using the minimum norm estimation (MNE) with the sLORETA approach (dipole orientation: unconstrained) applied to improve spatial accuracy.

### Hidden Markov modeling

Source-reconstructed data were analyzed using the OHBA Software Library (OSL v2.0.3; OHBA Analysis Group, 2017) and the HMM-MAR toolbox (Chetan et al., 2024; Vidaurre et al., 2016). Within this framework, covariance matrices were computed across the entire time course for each participant and regularized through principal component analysis (PCA)-based rank reduction. Source-space activity was then estimated using a beamformer approach across a 38-node cortical parcellation derived from the atlas by Colclough et al. (2015), following the methodology described by Toffoli et al. (2024); see also Quinn et al. (2018). Signal leakage was corrected using multivariate orthogonalization, and signal amplitude was estimated via the Hilbert transform. Functional brain states were identified across the concatenated datasets of all participants and conditions. The optimal number of states (K) was determined by computing the HMM for several state configurations (3, 4, 5, 6, 7, 8, and 9 states) and calculating the Bayesian Information Criterion (BIC) and Akaike information criterion (AIC) for each configuration, with lower values indicating a better trade-off between model complexity and quality of fit. These values were plotted graphically, and the “elbow point” was identified as the minimum number of states that best explained the variance in the data (Zucchini and MacDonald, 2009). Both parameters identified the configuration at six states as the optimal solution for the data. Hence, the six resulting states and their associated parameters (FO, LT, IT, and SR) considered for the statistical analyses.

To further assess the robustness of this model configuration, a split-half validation was performed by randomly dividing participants into two subgroups (*N* = 14 and *N* = 13) and re-running the HMM separately for each. This additional analysis yielded highly comparable state topographies and HMM parameters across subgroups, supporting the stability and reliability of the six-state solution. The resulting State’s topographies, FO distributions, and the correlations between the full dataset and each subgroup in terms of FO are reported in Supplementary Figures S1, S2 and Table S1.

### Statistical analysis

Statistical analyses were performed using JASP (version 0.19.3; JASP Team, 2024). The primary goal was to examine the HMM-derived metrics and their relation to behavioral performance. Fractional occupancy (FO) was investigated across conditions and participants using a repeated-measures ANOVA (rmANOVA) with a 3 (condition: naturalistic viewing, event, time) × 6 (number of states: 1, 2, 3, 4, 5, and 6) within-subjects design. State lifetimes (LT) and interval times (IT), which are calculated over the whole concatenated dataset, were investigated across states through separate ANOVA analyses with number of states as the within-subject factor. Switching rate (SR), which is computed across all states, was compared across the three conditions using a one-way ANOVA (Tang et al., 2012).

Behavioral data were analyzed more extensively in a separate study that examined EEG activity in the time–frequency domain across the three conditions (Santacesaria et al., 2025). In the present work, behavioral measures were considered only in relation to HMM-derived metrics, focusing on the 27 participants included in the EEG analyses. The metrics considered from both PM conditions consisted of accuracy and response times (RTs). In the event-based PM condition, accuracy was defined as the percentage of correctly detected PM cues. In the time-based PM condition, a response was considered correct if made within ±6 s of the target time (10% of the total 2-min interval), consistent with existing literature (Vanneste et al., 2016). Concerning the time-based PM condition, the number of time checks was also considered, both in terms of their total number and the number of “efficient time checks,” defined as those occurring within the 30 s preceding the target time (Hicks et al., 2000). A summary of participants’ behavioral performance is provided in Table 1.

**Table 1:** Mean performance measures across the event-based and time-based PM tasks.

	Accuracy [95% CI]	Response times (s)	Total number of time checks	Efficient time checks
Event-based PM task	96.471 [92.4, 100]	1.333 ± 0.514	–	–
Time-based PM task	71.765 [54.69, 88.84]	−0.871 ± 1.793	10.647 ± 3.707	1.941 ± 1.144

Accuracy is reported as the percentage of correct responses with 95% confidence intervals (CIs), response times are expressed as the average temporal distance (in seconds) from the target cues. In addition, for the time-based PM condition, the table includes the average number of time checks performed and the number of efficient time checks, defined as those occurring within the final 30 s preceding the target cue.

To investigate the relationship between behavioral performance and the dynamic properties of the hidden states identified by the HMM, Spearman’s rank correlations were calculated between behavioral measures (accuracy, RTs, total time checks, and efficient time checks) and HMM-derived metrics (FO and SR), separately for each condition, following the approach of Taghia et al. (2018). Significance levels were corrected for multiple comparisons using the false discovery rate (FDR) method (Benjamini and Hochberg, 1995).

## Results

### Markov states

**Figure 2:**
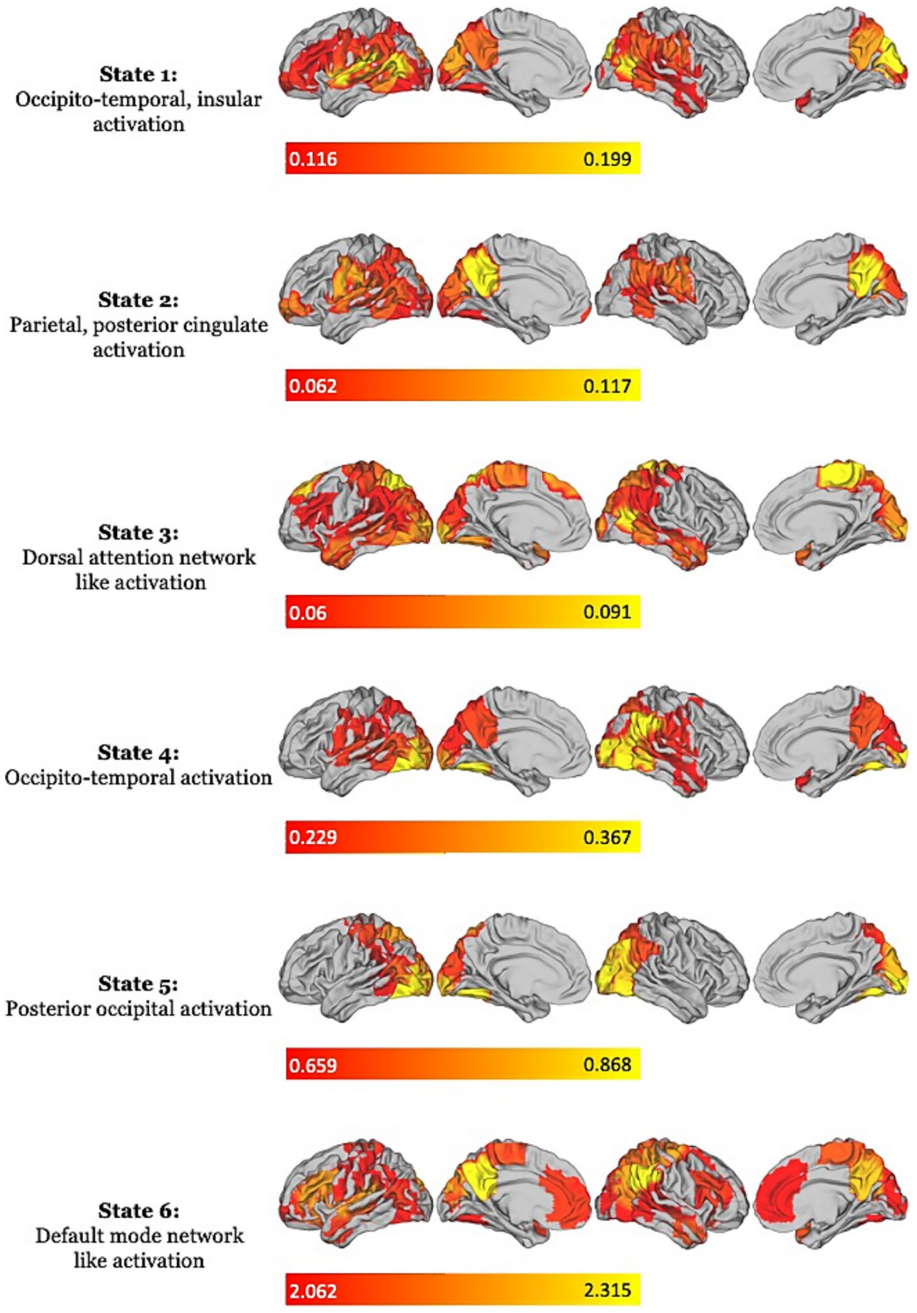
Visualization of the six Hidden Markov Model (HMM) states. For each state, the top 45% of the most active positive activations are plotted onto cortical space using the HCP Workbench GUI. Activation intensity is represented with a color gradient, ranging from red (weakest activations) to yellow (strongest activations). On the right, a label identifying the core regions/networks associated with each state.

**Figure 3:**
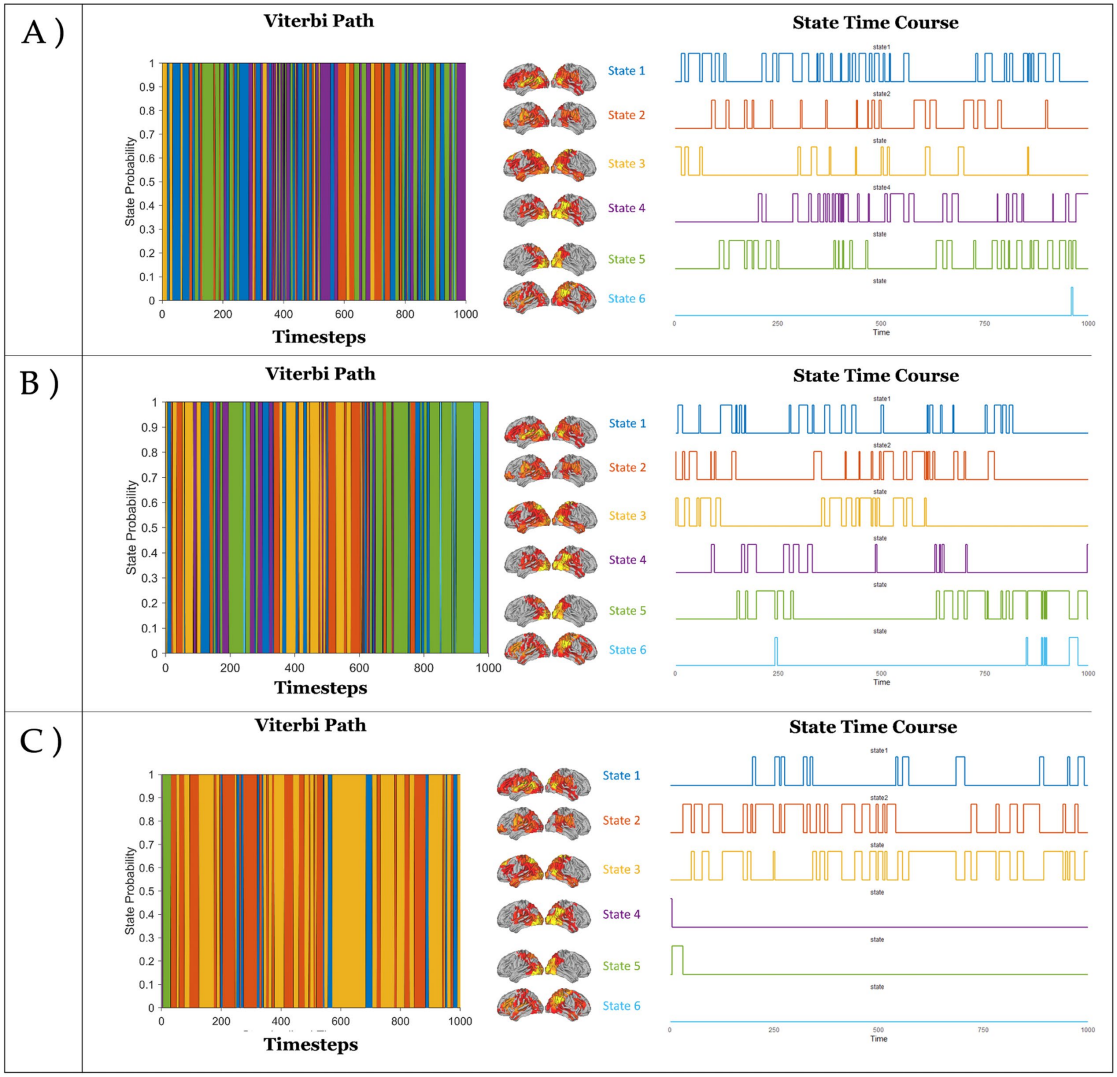
Temporal dynamics of the six HMM states across the three conditions: Naturalistic Viewing **(A)**, Event-based PM condition **(B)** and Time-based PM condition **(C)**. For each condition, a representation of 1000 timesteps (4 seconds sampled at 250 Hz) of the Viterbi Path (the maximum a posteriori sequence of states) is shown on the left. On the right, the time course of each state is visualized during the same 1000 timesteps.

The BIC and AIC analyses revealed that the six-state solution provided the best fit for the data, optimizing explained variance while maintaining the lowest level of complexity. Visual inspection further supported this choice, revealing that the six states captured distinct spatiotemporal patterns corresponding to specific neural regions, broadly resembling large-scale functional networks (Figure 2). Each state’s topography represented the average activation profile across parcels in the concatenated EEG dataset, encompassing all three conditions (naturalistic viewing, event-based, and time-based). State 1 was characterized by activations over occipital, temporal, and insular regions, while State 2 displayed a similar topography but with higher medial activation, mostly localized in the precuneus. State 3 exhibited a topography dominated by activity over frontal and parietal regions, encompassing regions such as the Frontal Eye Fields, Superior Parietal Lobule, and Middle Temporal Motor Area, typically associated with the DAN. States 4 and 5 primarily involved activity over posterior regions, including primary and secondary visual areas, the fusiform gyrus, and the temporoparietal junction. Finally, State 6 displayed a topography overlapping regions often associated with the DMN, including the medial prefrontal cortex, precuneus, and angular gyrus, as well as in areas associated with the VAN, such as the anterior cingulate cortex and temporoparietal junction. This state also showed activation in the primary motor cortex, along with the highest levels of activation over its parcels The range of activation for each state is illustrated in Figure 2, whereas Figure 3 presents the states’ activation probabilities and time courses across the three conditions, using a representative example of 1,000 time steps (approximately 4 s) extracted from each condition.

### Fractional occupancy, state lifetimes, interval times, and switching rate

**Figure 4:**
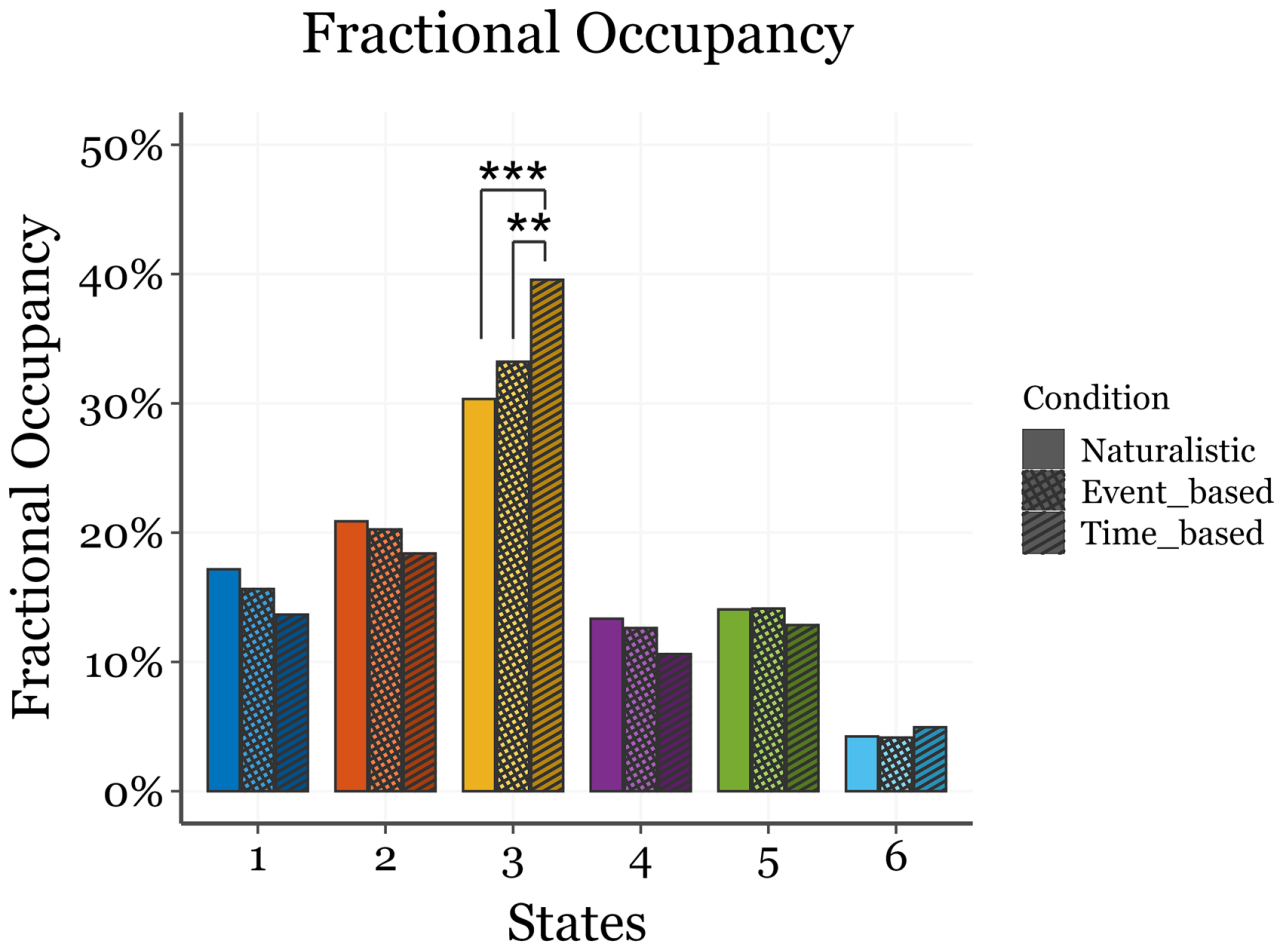
Comparison of Fractional Occupancy (FO) of the six states (horizontal axis) in the three conditions. Each state is represented by a specific color, displayed in different shades in the three conditions. Bars represent standard errors and asterisks indicate significant differences between conditions within the same state (*p_bonf_ < .05; **p_bonf_ < .01; *** *p*_bonf_ < .001).

The rmANOVA on FO revealed significant main effects of State [*F*(5, 135) = 49.586; *p* < 0.001; ηp^2^ = 0.756] and State*Condition [*F*(10, 270) = 5.307; *p* = 0.010; ηp^2^ = 0.249]. *Post-hoc* analyses with Bonferroni correction indicated that State 3, associated with activation over DAN regions, and State 6, associated with the DMN, significantly differed from all other states (Figure 4). Specifically, State 3 exhibited the highest FO across all conditions (time-based: 39.6%; event-based: 33.2%; naturalistic viewing: 30.3%), while State 6 displayed the lowest (~4% in all conditions). Participants spent significantly more time in State 3 during the time-based PM task compared to the other two conditions (*p*_bonf_ < 0.001). While there was also a difference between the event-based and the naturalistic viewing conditions, it did not survive the correction for multiple comparisons (*p*_bonf_ = 0.173).

**Figure 5:**
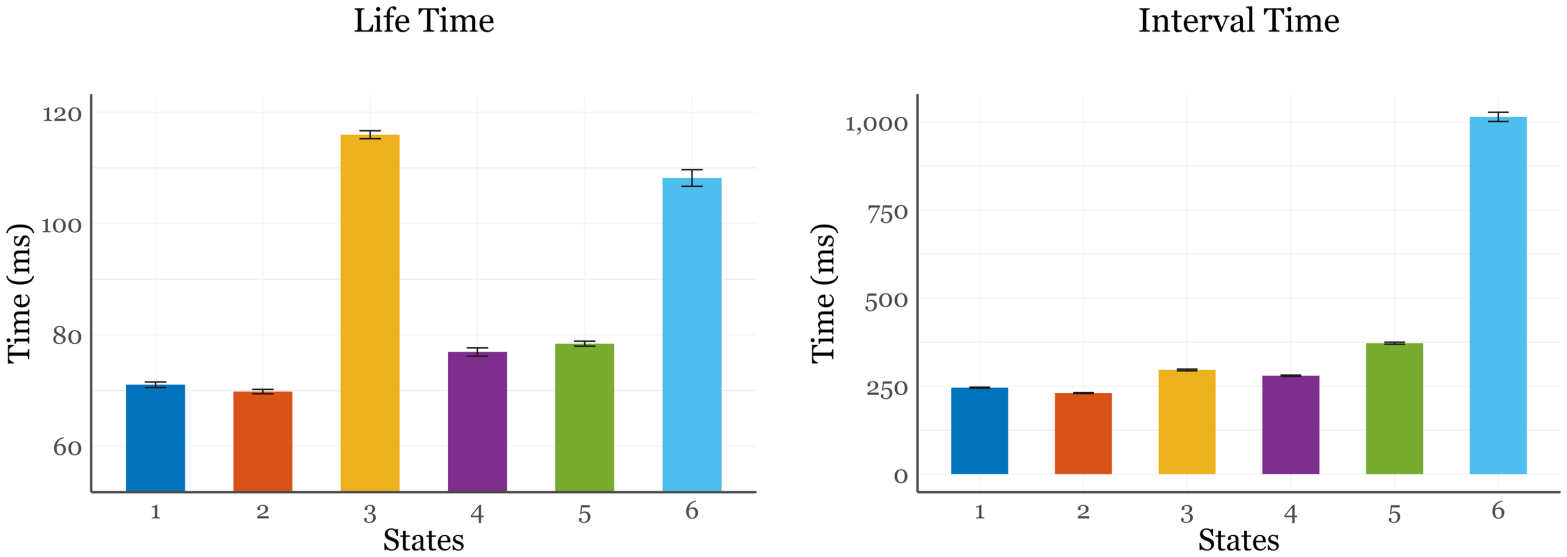
Visual representation of the State Lifetimes (left) and Interval Times (right) of the six states identified through HMM analysis. LT represents the average duration each state was visited before transitioning, whereas IT reflects the average interval time between successive occurrences of the same state.

Analysis of state LT revealed a moderate effect of State [*F*(5, 33,499) = 483.181; *p* < 0.001; ηp^2^ = 0.67], primarily driven by longer intervals spent in States 3 (116 ms) and 6 (108 ms) compared to the others, which ranged between 70 and 78 ms. IT showed a strong effect of State [*F*(5, 115,527) = 4689.916; *p* < 0.001; ηp^2^ = 0.169], with all states differing significantly from one another (*p*_bonf_ < 0.001). While the first five states had relatively similar ITs (ranging between 182 and 410 ms), the intervals between consecutive visits to State 6 were substantially longer (1,014 ms). Figure 5 displays LT (left) and IT (right) across the six states. Finally, the examination of switching rate (SR) revealed no significant effect of Condition [*F*(2, 54) = 0.318; *p* = 0.729; ηp^2^ = 0.013].

### Correlation between fractional occupancy, switching rate, and behavioral measures

Correlational analyses were conducted to examine potential relationships between HMM metrics—FO and SR in particular—and behavioral performance. No significant associations emerged between FO or SR and accuracy, response times, or total number of time checks. Arguably, the absence of correlations between accuracy and HMM metrics may be due to the low number of trials (five in both PM tasks) and the ceiling effect in participants’ performance in the Event-based PM task (mean accuracy = 96.5%, confidence interval [92.4, 100]). On the other hand, the number of efficient time checks (those occurring within 30 s from the target cue) showed strong correlations with several HMM measures (Table 2). Specifically, efficient time checks positively correlated with FO of States 3 and 6 in the time-based PM task (Rho = 0.704, *p*_FDR_ = 0.011 and Rho = 0.636, *p*_FDR_ = 0.026, respectively), suggesting that participants who spent more time in these neural states tended to monitor time more effectively. Efficient time checks also correlated with SR, selectively in the two PM conditions: more accurate time monitoring was associated with a lower frequency of state switching in the time-based (Rho = −0.690, *p*_FDR_ = 0.045) and event-based (Rho = −0.598, *p*_FDR_ = 0.014) PM tasks, but not with in the naturalistic viewing condition (Rho = −0.363, *p*_FDR_ = 0.302).

**Table 2:** Correlation map between efficient time checks (a measure of time estimation performance), fractional occupancy (FO) of the two states associated with activity over DAN (State 3) and DMN (State 6) regions, and switching rate (SR) in the three experimental conditions (naturalistic viewing, event-based PM task, time-based PM task).

	Time checks	State 3 FO naturalistic	State 3 FO event	State3 FO time	State 6 FO naturalistic	State 6 FO event	State 6 FO time	SR naturalistic	SR event
Time checks	—								
State 3 FO naturalistic	0.410	—							
State 3 FO event	0.511	0.831^***^	—						
State3 FO time	0.704^*^	0.475	0.679^*^	—					
State 6 FO naturalistic	0.327	0.941^***^	0.740^**^	0.397	—				
State 6 FO event	0.587	0.868^***^	0.919^***^	0.674^*^	0.799^***^	—			
State 6 FO time	0.636^*^	0.549	0.718^**^	0.892^***^	0.500	0.752^**^	—		
SR naturalistic	−0.363	−0.782^**^	−0.784^***^	−0.490	−0.792^**^	−0.789^**^	−0.676^*^	—	
SR event	−0.598^*^	−0.485	−0.578	−0.324	−0.387	−0.650^*^	−0.426	0.363	—
SR time	−0.690^*^	−0.532^*^	−0.652^*^	−0.706^*^	−0.436	−0.650^*^	−0.779^**^	0.610^*^	0.456

Significance (corrected for multiple comparisons) is represented as follows: *p < 0.05, **p < 0.01, ***p < 0.001.

## Discussion

In this study, we designed a pseudo-naturalistic PM paradigm simulating everyday activities to investigate the neural correlates of prospective remembering in realistic scenarios. High-density EEG data were collected continuously during three conditions: a baseline condition, an event-based PM task, and a time-based PM task. These data were projected to the source level using individual MRI scans and analyzed using a hidden Markov modeling (HMM) approach. This method identified distinct patterns of brain activity (i.e., states), each with specific and temporal properties. By examining states’ topographies and these HMM-derived features, we explored the dynamics associated with intention maintenance, external monitoring, and retrieval, offering a novel methodological and statistical framework to study PM processes in settings that more closely mimic real-life conditions.

The HMM decomposition revealed that the neurophysiological data collected in the three conditions were best explained by six discrete brain states, each defined by distinct patterns of cortical activation, some of which displaying topography broadly paralleling well-characterized functional networks (Yeo et al., 2011; Uddin et al., 2019). This finding reinforces the view of PM as a non-unitary process, supported by the dynamic interplay of multiple brain networks that fluctuate in response to cognitive demands and features of the PM task (Cona et al., 2015; McDaniel et al., 2015). The six identified states displayed topographies either associated with PM mechanisms or ongoing task-related activity (movie watching). Specifically, State 1 was characterized by activations over occipital, temporoparietal, and insular regions, possibly corresponding to activity associated with the Visual Network (VN) and the VAN. State 2 exhibited a similar configuration but with marked activation of the precuneus, a key hub of the DMN. State 3 exhibited robust activations in regions associated with the DAN, including the inferior parietal lobule, the V5/MT complex, and the frontal eye fields. States 4 and 5 were dominated by activity in occipital and inferotemporal regions such as the primary visual cortex and fusiform gyrus, which are parts of the VN and are associated with visual processing. In contrast, State 6 was marked by strong activation over DMN regions, including the angular gyrus, precuneus, and medial prefrontal cortex, alongside VAN regions such as the anterior cingulate cortex and supramarginal gyrus. Notably, this state also showed significant activation in somatomotor regions—a pattern not present in the other states.

As discussed in the Introduction, the DAN and VAN play fundamental roles in PM, supporting cue monitoring and intention retrieval, respectively (Cona et al., 2015). The DMN, on the other hand, is more implicated in intention maintenance and, together with the VAN, contributes to intention retrieval from memory (Cona et al., 2020; Vicentin et al., 2025). Within this framework, the pattern elicited in State 3 likely reflects sustained monitoring during the maintenance phase, given the involvement of core DAN regions and its fronto-temporal spread. This interpretation aligns closely with sensor-level findings from the same dataset (Santacesaria et al., 2025), which revealed enhanced fronto-temporal high-beta power during the time-based PM condition—an effect consistent with increased top-down attentional engagement. In contrast, State 6 displayed a topography suggestive of intention retrieval and, given the inclusion of somatomotor areas and its higher activation levels, execution processes. Regarding the other states, the patterns dominated by occipital activity (particularly States 4 and 5) can be attributed to the ongoing movie-watching task, which likely induced sustained activation in visual regions (Chang et al., 2015). The predominance of occipito-parietal activations in the event-based and Naturalistic Viewing conditions further echoes both the theta–alpha modulations observed at the sensor level (Santacesaria et al., 2025) and previous evidence linking posterior alpha power to attentional orienting (Madore et al., 2020; Tran et al., 2025). It is worth noting, however, that while these studies examined specific frequency bands, our HMM analysis encompasses the 1–30 Hz range, providing a broader view of how neurophysiological activity dynamically reconfigure during prospective remembering.

To further elucidate the functional significance of the six states, we examined the HMM-derived temporal metrics—FO, LT, IT, and SR. These indices provided insights into the temporal dynamics of brain state engagement and their relationship with PM processes. Namely, participants exhibited significantly higher FO in State 3 (the DAN-dominant state) during the PM conditions, particularly in the time-based PM task, compared to the naturalistic viewing condition. Given that FO reflects the proportion of time spent in a given state, this pattern suggests a prominent role of State 3 in sustained attentional control and top-down monitoring processes required for intention maintenance—especially when time estimation is involved. Importantly, FO differences in State 3 reached statistical significance only between the time-based PM task and the other two conditions; the difference between the event-based PM and the naturalistic viewing conditions did not survive correction for multiple comparisons. This suggests that the event-based PM task, which involved detecting the sporadic occurrence of a visual cue, may have been less demanding in terms of external monitoring (Cona et al., 2012; Oksanen et al., 2014). Additionally, State 3 included activations in non-DAN regions, such as the cuneus and superior temporal gyrus, which are implicated in time estimation processes (Wiener et al., 2010), making them selectively involved in time-based PM tasks (Gonneaud et al., 2014). In contrast, event-based PM tasks showed greater activation in occipito-parietal regions (States 4 and 5), overlapping with areas engaged during movie watching and visual processing (VN and VAN; Fishell et al., 2019). Although these visually driven states likely reflected perceptual engagement, they could also interact with PM-related dynamics, as event-based tasks tend to rely on similar occipital and parietal regions (Gonneaud et al., 2014). Effective prospective remembering may thus depend on the transient modulation of these perceptual states, allowing task-relevant control networks (e.g., DAN- and DMN-related) to guide behavior—an open question that future research should address.

States 3 and 6 displayed significantly longer LT compared to the other states, suggesting greater stability in their activation patterns. Additionally, State 6 exhibited significantly longer IT between consecutive visits, potentially reflecting its role in sporadic, stimulus-driven attentional shifts. Given their associations with DAN and DMN regions, respectively, these findings highlight the complementary roles of States 3 and 6 in PM, with State 3 likely involved in intention maintenance, and State 6—given its topography and dynamics—possibly supporting intention retrieval. This interpretation aligns with previous research emphasizing the complementary roles of the DAN and DMN in monitoring and retrieval processes (Cona et al., 2020; Vicentin et al., 2025). Moreover, the continuous alternation among transient neural states and the relatively brief lifetime of each visit suggest that prospective remembering may be shaped by ongoing fluctuations in attentional engagement. Such dynamics resonate with the idea that attention operates in rhythmic “waves,” modulating access to mnemonic representations (Madore et al., 2020; Schwartz et al., 2025). In this framework, rapid transitions between DAN- and DMN-dominant states may index oscillations between externally oriented monitoring and internally oriented retrieval, reflecting how variations in attentional intensity and selectivity dynamically influence the maintenance and retrieval of intentions over time.

Correlations between behavioral measures and HMM outputs further support the association between States 3 and 6 and PM processing. Specifically, FO in States 3 and 6 during the time-based PM task positively correlated with the number of efficient time checks, suggesting that increased engagement in these states was associated with more accurate time estimation (Cabral et al., 2017; Cornblath et al., 2020). Moreover, SR in the two PM tasks (but not in the naturalistic viewing condition) negatively correlated with efficient time checks, indicating that lower switching rates and greater neural stability were linked to better PM performance. This finding is consistent with previous research demonstrating negative relationships between switching rate and task performance (Matos et al., 2020; Taghia et al., 2018; Zdorovtsova et al., 2023).

By integrating HMM analysis with a pseudo-naturalistic paradigm, this study offers a multidimensional framework for investigating PM-related neural dynamics. Unlike traditional laboratory paradigms that rely on repetitive trials and artificial stimuli, our design aimed to mimic real-world PM scenarios, such as monitoring meal preparation while watching TV. This approach enhances ecological validity and captures PM processes over extended time scales, providing a more accurate reflection of the neural correlates of intention maintenance and retrieval in everyday life (Rummel and Kvavilashvili, 2019). Hopefully, this study will contribute to bridging the gap between controlled laboratory paradigms and real-life PM contexts, paving the way for new research questions and methods to explore the neural mechanisms of prospective remembering in more ecological settings and more compromised clinical populations.

## Limitations and future directions

Despite these contributions, several limitations warrant consideration. First, although individual MRI-based source modeling improves spatial accuracy, the spatial precision of EEG remains limited relative to fMRI. Consequently, the labeling of states based on their correspondence with canonical networks (e.g., State 3 as DAN-like) is necessarily inferential and should be interpreted with caution. Second, while the sample size was adequate for EEG studies, larger and more demographically diverse populations—including children, older adults, and clinical groups—are essential to generalize findings. Currently, data are being collected from a healthy older adults (aged 65 and above) group and individuals with Parkinson’s disease, given the link between this pathology and PM deficits (Ramanan and Kumar, 2013). Third, while our pseudo-naturalistic paradigm enhances ecological validity, it also introduces variability in engagement and cognitive load. Future studies could incorporate additional measures, such as eye-tracking or self-reports, to better account for these factors. Third, although the HMM is a powerful tool for analyzing neural dynamics, its outputs are sensitive to model parameters and the quality of the input data (Quinn et al., 2019; Vidaurre et al., 2016). For example, recent evidence suggests that activity in the DMN may be more accurately captured when focusing on alpha-band oscillations (8–13 Hz), rather than broadband signals (Marino et al., 2019). This highlights the importance of conducting more targeted analyses to improve sensitivity to specific networks. Accordingly, future research could benefit from integrating complementary methods—such as dynamic functional connectivity—to enhance the robustness and interpretability of HMM-derived results.

## Conclusion

This study provides novel insights into the dynamic neural mechanisms underlying PM by integrating a pseudo-naturalistic paradigm with HMM analysis. Our findings underscore the complementary roles of the DAN and the DMN in prospective remembering, the former implied in sustained monitoring during intention maintenance, the latter engaged intermittently to support intention retrieval from memory. The results also emphasize the value of ecologically valid paradigms in cognitive neuroscience. By narrowing the gap between controlled experiments and real-world PM, this study offers a methodological framework that advances our understanding of how attentional and mnemonic systems interact to support the ability to “remember to remember” in everyday life.

## Data Availability

The raw data supporting the conclusions of this article will be made available by the authors, without undue reservation.
