# Supplementary material for: Decoding the neural dynamics of everyday prospective remembering: a hidden Markov model approach
Source: Front Hum Neurosci. 2026 Feb 4;19:1686657. doi: 10.3389/fnhum.2025.1686657 (PMC12914720; doi:10.3389/fnhum.2025.1686657)
Supplement: Supplementary file 1 [file Table_1.pdf]

Table S1. Correlation map between Fractional Occupancy (FO) analysis of the total dataset (bottom row) and the two subgroups obtained with the split-half analysis. Pearson Correlation analyses were performed on the values of FO of each state, separately for condition (Naturalistic Viewing, Event-based PM task, Time-based PM task). Significance (corrected for multiple comparisons) is represented as follows: * $p < .05$, ** $p < .01$, *** $p < .001$.

	**Naturalistic Viewing**		**Event-Based PM task**		**Time-Based PM Task**	
	**First Half**	**Second Half**	**First Half**	**Second Half**	**First Half**	**Second Half**
**First Half**	—	0.680***	—	0.706***	—	0.544***
**Total Dataset**	0.871***	0.680***	0.832***	0.711***	0.899***	0.579***
