# Supplementary figures and images for: Decoding the neural dynamics of everyday prospective remembering: a hidden Markov model approach
Source: Front Hum Neurosci. 2026 Feb 4;19:1686657. doi: 10.3389/fnhum.2025.1686657 (PMC12914720; doi:10.3389/fnhum.2025.1686657)

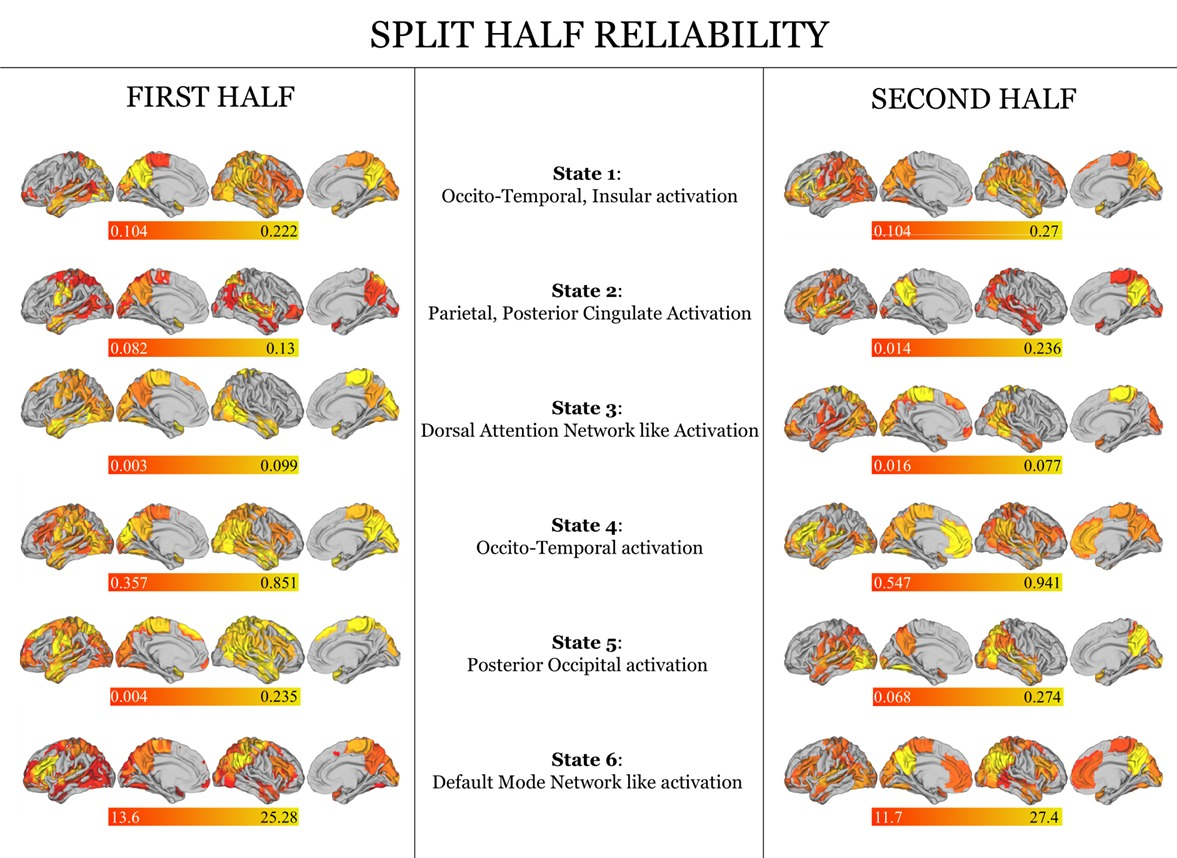

Supplement: Supplementary file 2 [file Image_1.tiff]

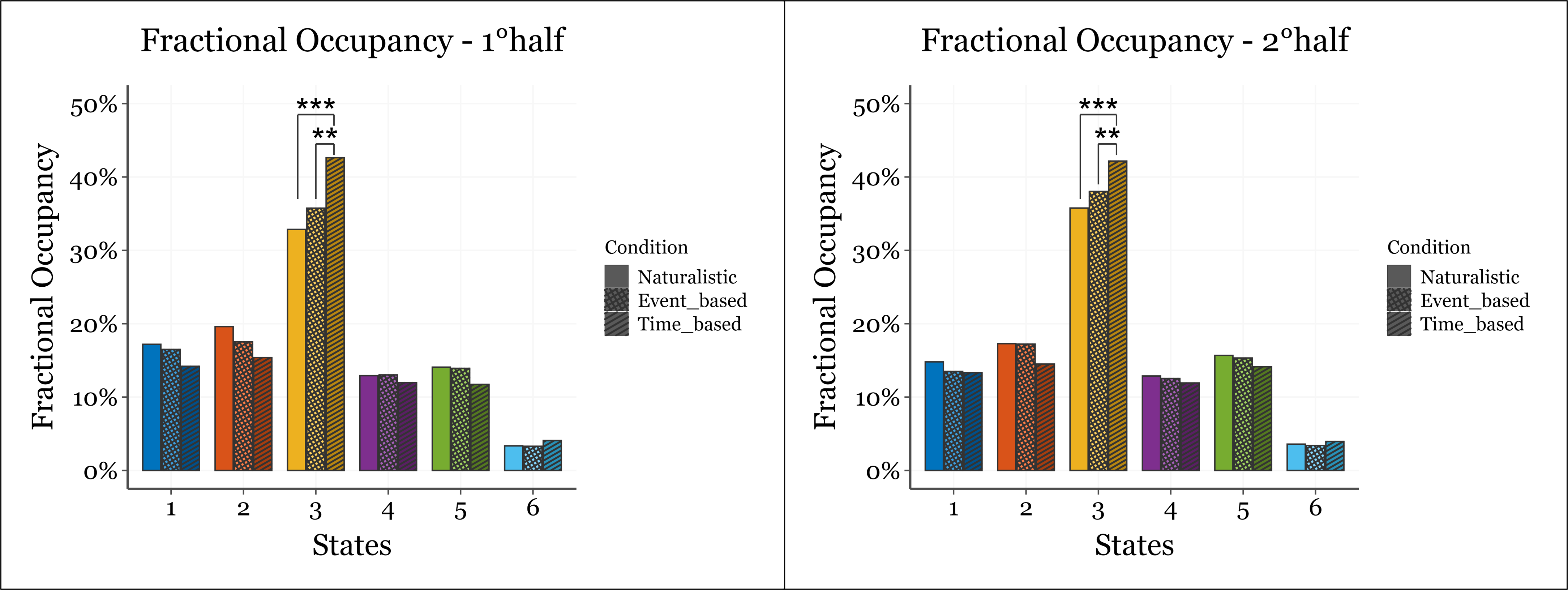

Supplement: Supplementary file 3 [file Image_2.tiff]
